# Application of PET/MRI in Gynecologic Malignancies

**DOI:** 10.3390/cancers16081478

**Published:** 2024-04-12

**Authors:** Sheida Ebrahimi, Elin Lundström, Summer J. Batasin, Elisabeth Hedlund, Karin Stålberg, Eric C. Ehman, Vipul R. Sheth, Negaur Iranpour, Stephane Loubrie, Alexandra Schlein, Rebecca Rakow-Penner

**Affiliations:** 1 Department of Radiology, University of California San Diego, La Jolla, CA 92093, USA; 2 Department of Surgical Sciences, Radiology, Uppsala University, 751 85 Uppsala, Sweden; 3 Center for Medical Imaging, Uppsala University Hospital, 751 85 Uppsala, Sweden; 4 Department of Women’s and Children’s Health, Uppsala University, 751 85 Uppsala, Sweden; 5 Department of Radiology, Mayo Clinic, Rochester, MN 55905, USA; 6 Department of Radiology, Stanford University, Palo Alto, CA 94305, USA; vsheth@stanford.edu (V.R.S.);; 7 Department of Bioengineering, University of California San Diego, La Jolla, CA 92093, USA

**Keywords:** PET/MRI, gynecological malignancy, cervical cancer, endometrial cancer, ovarian cancer, vaginal cancer

## Abstract

**Simple Summary:**

This article reviews the value of Positron Emission Tomography/Magnetic Resonance Imaging (PET/MRI) in evaluating female pelvic cancers. It also provides a comparative analysis of PET/MRI with other imaging modalities in the context of female pelvic malignancies and outlines their respective strengths and limitations. The aim of this narrative review is to introduce to clinicians up and coming technology and how it may be valuable to their assessment of female pelvic cancers.

**Abstract:**

The diagnosis, treatment, and management of gynecologic malignancies benefit from both positron emission tomography/computed tomography (PET/CT) and MRI. PET/CT provides important information on the local extent of disease as well as diffuse metastatic involvement. MRI offers soft tissue delineation and loco-regional disease involvement. The combination of these two technologies is key in diagnosis, treatment planning, and evaluating treatment response in gynecological malignancies. This review aims to assess the performance of PET/MRI in gynecologic cancer patients and outlines the technical challenges and clinical advantages of PET/MR systems when specifically applied to gynecologic malignancies.

## 1. Introduction

Gynecological pelvic cancers, including cervical, endometrial, ovarian, vaginal, and vulvar cancers, account for approximately 15% of cancers diagnosed in women worldwide [1,2]; in the United States, 114,810 new cases and 34,020 deaths are estimated for 2023 [3]. When staging gynecological pelvic cancers, the local tumor extent, metastases to nearby lymph nodes, and distant metastases each need to be characterized. This usually requires multiple imaging modalities, including both PET/CT and MRI. PET/CT characterizes both local tumor extent and distant metastasis [4,5,6]. MRI provides important soft-tissue detail and information on the local–regional extent of disease. PET/MRI has been increasingly utilized in clinical practice and offers advantages over PET/CT or MRI alone [7]. These advantages include combining each modalities’ strength into one exam, with direct anatomic correlation such as improved soft-tissue contrast, co-registration of metabolic and MRI anatomic images, and reduced radiation exposure.

PET/MRI demonstrates higher sensitivity and specificity than either PET/CT or MRI alone in staging, detecting gynecologic cancer recurrence, and the assessment of post-treatment response [8,9,10,11,12,13,14].

This review outlines the current and potential utilization of PET/MR for gynecological cancer imaging and how the technology can be applied in diagnosing, staging, restaging, and monitoring treatment responses for female gynecological cancer. A relevant overview of the PET/MR technique, including systems, biomarkers, advantages, challenges, and prospects, is provided. 

## 2. PET/MRI

Cross-sectional imaging provides helpful information for more accurate diagnosis, staging, treatment planning, and monitoring of gynecologic malignancies (Table 1), each technique with its benefits and pitfalls [15]. The first clinical CT scanner was installed in 1971 [16]. By the early 1980s, 1.5 T MRI scanners became clinically available [17]. PET/CT and PET/MRI became clinically available in the mid-1990s and 2010s, respectively [18].

The combination of whole-body PET and MRI, with targeted MRI of the specific organ of interest, offers a unified solution for local staging and detection of distant metastases. Systems are available in three main designs: tri-modality, sequential, and integrated (Table 2). Of the three designs, integrated PET/MR offers a comprehensive acquisition where the MRI and PET can be acquired simultaneously. However, not all facilities offer the integrative design, and similar diagnostic information can be provided from the tri-modality and sequential designs. The main benefit of the integrative approach regards image registration and increased convenience for the patient for a single diagnostic exam.

### 2.1. Oncologic PET Tracers and Patient Preparation

The primary radiotracer in gynecologic oncology PET/MRI (and PET/CT) is ^18^F-fluorodeoxyglucose (FDG), which targets increased glucose metabolism in malignancy. Some research studies have highlighted the benefits of prostate-specific membrane antigen (PSMA)-targeted ^18^F-DCFPyL and ^68^Ga-FAPI-04 (fibroblast activating protein inhibitor) in diagnosing and assessing the treatment of ovarian cancer [48,49]. Additionally, ^68^Ga-FAPI-04, ^68^Ga-RGD (arginine–glycine–aspartic acid peptide) and ^18^F-EF5 have shown promise in diagnosing and planning radiation treatment for cervical cancer [50,51,52].

Proper patient preparation enhances the interpretative accuracy of PET/MRI. Minimizing the influence of metabolic changes and peristalsis are key objectives. The primary radiotracer in the oncologic PET/MRI of the female pelvis is FDG. Metabolic activity can be influenced by several factors, including diet, extreme physical activity, trauma, medications, infection/inflammation, and environmental temperature [45]. Therefore, a consideration of these factors when performing a PET/MRI may be helpful to ensuring the quality and reliability of results. FDG-PET imaging is optimized by patients fasting for at least four hours before injecting the radiotracer to achieve a target blood glucose level below 160 mg/dL. To improve image quality, an antiperistaltic agent such as butylscopolamine bromide (Buscopan) or glucagon can be administered [53,54]. Patients may undergo PET scanning one hour after the radiotracer injection.

### 2.2. Quantitative Imaging Biomarkers 

Both PET scanning and MRI offer quantitative biomarkers (Table 3) that individually provide valuable information about the region of interest, including cellular density and metabolic activity. The main quantitative biomarker for MRI is derived from diffusion-weighted imaging (DWI).

Several pathological prognostic variables including tumor stage and overall survival (OS) are correlated either individually or in combination with PET/MRI biomarkers. According to a prospective study by Shih et al., SUV_max_ and ADC_min_ are both independent predictors of progression-free survival (PFS) and OS [55]. They also found MTV/ADC_min_ is the strongest predictive biomarker for tumor stage, and ADC_min_ is significantly lower in advanced cancer stages (≥IB3), while MTV, TLG, MTV/ADC_min_, and TLG/ADC_min_ are higher. Furthermore, Steiner et al. indicated the potential predictive power of SUV_max_/ADC for determining nodal status by reporting higher tumor SUV_max_/ADC in patients with metastatic pelvic lymph nodes [56].

### 2.3. Advantages of PET/MRI

PET/MRI has several advantages compared with alternative stand-alone imaging modalities and PET/CT. First, it provides complementary information from both PET and MRI, resulting in improved diagnostic accuracy for detecting pelvic malignancies. Second, PET/MRI reduces ionizing radiation exposure compared with PET/CT, making it a preferable option for patients requiring multiple follow-up examinations or those who are more sensitive to radiation exposure, such as children or women of childbearing age [57]. Third, PET/MRI shows higher reader diagnostic confidence in discriminating between benign and malignant lesions compared with PET/CT in cases of recurrent female pelvic malignancies [8]. PET/MRI enhances TNM staging, allowing a more accurate evaluation of the primary tumor locoregional extent, lymph node involvement, and metastasis compared with PET/CT [58]. Another advantage of PET/MRI is its ability to detect liver metastasis with higher accuracy compared with PET/CT [59,60]. Based on the study conducted by Gardner et al., the liver metastasis rates for stage IV ovarian, uterine, and cervical cancers were 57%, 22%, and 16%, respectively [61]. MRI is more sensitive to liver lesions than CT, and this allows for more accurate detection and differentiation between benign and malignant lesions [62]. Consequently, PET/MRI offers higher lesion conspicuity and diagnostic confidence compared with PET/CT for the characterization of liver lesions [60].

**Table 3:** Quantitative biomarkers.

Biomarker	Description	Clinical Interpretation
**PET Scan**		
SUV (Standardized Uptake Value)	Measure the uptake of the radioactive tracer in a specific region of interest (ROI) to assess the activity and metabolism of tissues SUV = Tracer concentration in ROI (kBq/mL)/Injected dose per body weight (kBq/g)	Inversely correlated with ADC [63,64,65,66,67,68,69,70,71,72,73] A higher SUV indicates higher metabolic activity in the ROI
SUV_mean_ (Mean Standardized Uptake Value)	Calculating the average tracer uptake in the selected ROI A comprehensive assessment of the overall tracer uptake within the ROI, useful for areas with varying tracer uptake (e.g., tumors)	Monitoring treatment response: a decrease in SUV from baseline indicates metabolic response to treatment [37] Prognosis: Overall survival is better in metabolic responders compared with metabolic non-responders [37]
SUV_max_ (Maximum Standardized Uptake Value)	Indicating the highest level of tracer uptake within a defined ROI Notable inverse correlation with ADC_min_ [55,74]	Diagnosis and staging: distinguish malignant (higher SUV_max_) and benign adnexal lesions [75] Treatment planning: Higher SUVmax values may indicate a more aggressive tumor [68] Monitoring treatment response: changes in SUV_max_ and especially the percent change value may have the potential to predict response to chemotherapy or chemoradiotherapy [36,38,76] Prognosis: changes in SUV_max_ predict the patient outcomes, disease recurrence, PFS [36,76,77]
MTV (metabolic tumor volume)	The metabolically active volume of the tumor (i.e., the portion of the tumor with a high SUV)	Staging: baseline MTV is a predictor of tumor characteristics such as MI and cervical stromal invasion, and lymph node metastasis; it is higher in cases with lymph node metastasis compared with those without such a metastasis Treatment planning: helps in determining the appropriate dosage and target volume for radiation treatment, ensuring that the radiation is delivered precisely to the areas containing tumor cells [78] Monitoring treatment response: the percentage of post-treatment changes in MTV is associated with the overall tumor response [35] Prognosis: the baseline MTV and the percentage of changes in MTV are predictive factors for OS, and PFS, recurrence [35,77,79]
TLG (Total Lesion Glycolysis)	provides a more comprehensive measure of tumor activity than SUV_max_ or SUV_mean_ alone TLG = SUV_mean_ × MTV	Staging: baseline TLG is a predictor of tumor characteristics, such as MI and cervical stromal invasion, and lymph node metastasis [77,80] Treatment planning: useful for radiation therapy planning by comprehensive assessment of the tumor burden [78] Monitoring treatment: change in TLG after treatment may have the potential to predict response to treatment [39,79] Prognosis: baseline TLG is prognostic factor of OS and PSF [39,77,78,79,81]
**DWI**		
ADC (Apparent Diffusion Coefficient)	Provides valuable information about tissue microstructure and cellular integrity [63,64,65,66,67,68,69,70,71,72,73] Inversely correlated with SUV	Helpful in differentiating between benign and malignant lesions, assessing tumor aggressiveness, and monitoring treatment response
ADC_min_ (Minimum Apparent Diffusion Coefficient)	Represents the region with the most restricted diffusion or the highest tumor cellularity Notable inverse correlation with SUV_max_ [67,74]	Diagnosis and staging: malignant tumors and regions with high cellular density tend to have lower ADC values, while benign or necrotic regions have higher ADC values Monitoring treatment: a decrease in ADC_min_ values after therapy can indicate a positive treatment response [55] Prognosis: independent predictor of OS [55]

## 3. Applications to Gynecologic Cancers

### 3.1. Cervical Cancer

**Figure 1:**
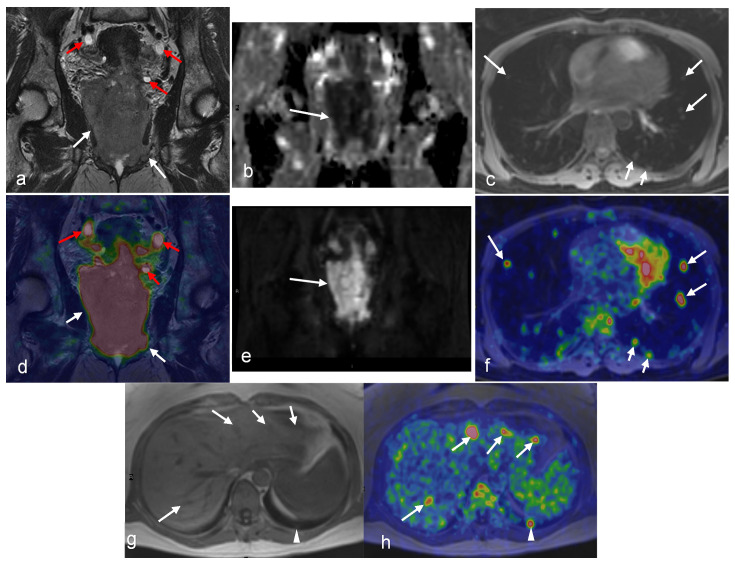
A 49-year-old woman with stage IVB cervical cancer. Both the coronal T2-weighted image (**a**) and fused PET/MRI (**d**) demonstrate the tumor with parametrial involvement and almost the entire vaginal vault (white arrows in (**a**,**d**)). There is also involvement of the bladder, ovaries, and proximal ureters (not imaged). Bilateral hydronephrosis is partially visualized (red arrows in (**a**) and (**d**)). The mass demonstrates diffusion restriction on the ADC map (arrow in (**b**)) and bright signal on the DWI (arrow in (**e**)). Axial Dixon water MRI (**c**) and axial-fused PET/MRI (**f**) show lung metastasis (arrows). Liver metastasis (arrows) and additional lung metastasis (arrowhead) are demonstrated in axial Dixon in-phase MRI (**g**) with high FDG uptakes on axial-fused PET/MRI (**h**). (Courtesy of Elisabeth Hedlund, MD, Håkan Ahlström, MD, and Björg Jónsdóttir, MD, PhD, Uppsala University, Uppsala, Sweden).

**Figure 2:**
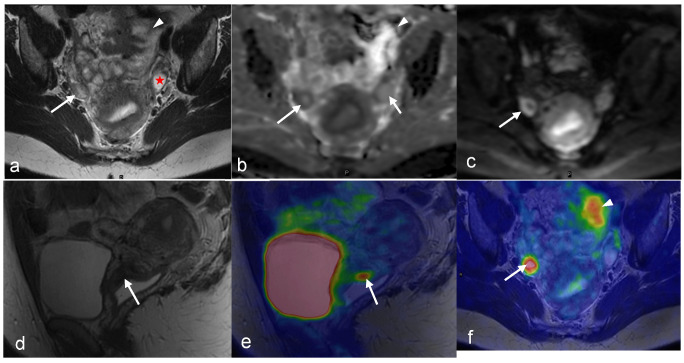
A 43-year-old woman with stage IB1 cervical cancer found to have a corpus luteum cyst in her right ovary. The cyst is visualized as a peripherally low-intensity structure with central high intensity (arrow in (**b**)) on ADC map and a peripherally hyperintense structure with low central intensity (arrow in (**c**)) on the axial diffusion-weighted image. The corresponding FDG uptake (arrow in (**f**)) on the fused PET/MRI is determined to be benign. In addition, the benign ovarian cyst in the left ovary is seen (star in (**a**)) with no pathological FDG uptake. The arrowhead in (**a**,**b**,**f**) shows part of the left superior corner of the bladder with corresponding FDG uptake in the urine. Sagittal T2-weighted MRI (**d**) and sagittal-fused PET/MRI (**e**) show the cervical mass (arrow in (**d**,**e**)). (Courtesy of Elisabeth Hedlund, MD, Håkan Ahlström, MD, and Björg Jónsdóttir, MD, PhD, Uppsala University, Uppsala, Sweden).

**Figure 3:**
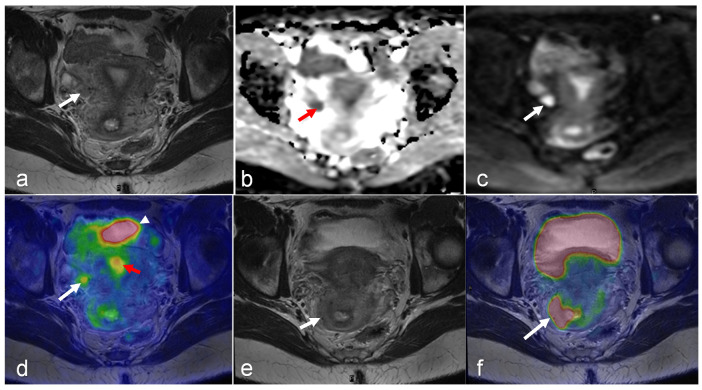
A 22-year-old woman with stage IB2 cervical cancer clinically or stage IIIC1 cancer as determined via PET/MRI due to a metastatic lymph node. The metastatic lymph node (arrow) is hypointense on the axial T2-weighted MRI (**a**); low signal on the ADC map (**b**); high signal on the DWI (**c**), with FDG uptake on fused PET/MRI (**d**) images. Physiologic FDG uptake in bladder (arrowheads in (**d**)) and endometrium (red arrow in (**d**)) can be seen. Both axial T2-wighted MRI (**e**) and axial-fused PET/MRI (**f**) show the cervical tumor with a suspicious irregular right margin (arrow in (**e**)) with pathologic FDG uptake interpreted as parametrial invasion (arrow in (**f**)). (Courtesy of Elisabeth Hedlund, MD, Håkan Ahlström, MD, and Björg Jónsdóttir, MD, PhD, Uppsala University, Uppsala, Sweden).

**Figure 4:**
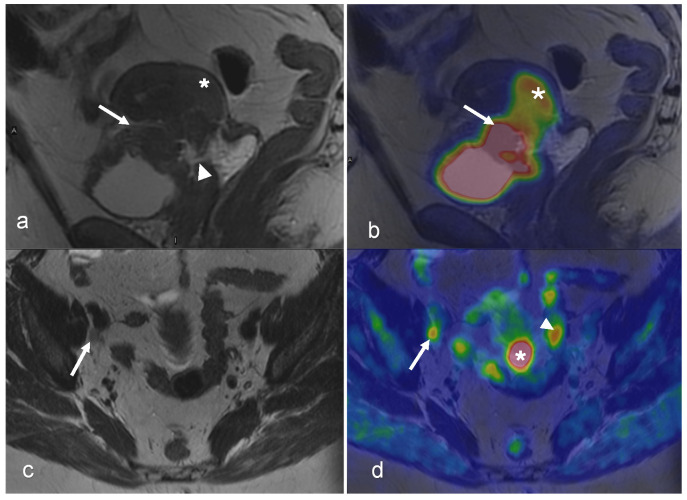
A 54-year-old woman with squamous cell carcinoma of the cervix, FIGO stage IVA. Sagittal T2-weighted MRI (**a**) shows a 43 mm tumor (star) with indications of invasion into the upper vagina and bladder (arrow) with suspected vesicovaginal fistula (arrowhead). Sagittal-fused PET/MRI (**b**) shows the FDG uptake of the tumor (star) and bladder wall (arrow). Both axial T2-weighted image (**c**) and axial-fused PET/MRI (**d**) demonstrate an 8 mm lymph node with irregular margins and pathologic FDG uptake (arrow in (**c**,**d**)). On axial-fused PET/MRI, (**d**) there is pathologic FDG uptake corresponding to the cervical tumor (star in (**d**)), which is difficult to distinguish on the axial T2-weighted image. Physiological FDG uptake in the bowel is seen on axial-fused PET/MRI (arrowhead in (**d**)). (Courtesy of Elisabeth Hedlund, MD, Håkan Ahlström, MD, and Björg Jónsdóttir, MD, PhD, Uppsala University, Uppsala, Sweden).

MRI and FDG-PET/CT are commonly used in the staging of invasive cervical cancer, characterizing local and distant disease, and predicting the likelihood of survival [82,83] (Figure 1, Figure 2, Figure 3 and Figure 4). Several studies attest to the overall benefits of PET/MRI compared with other modalities [56,84,85]. One retrospective study demonstrated that both tri-modality PET/MRI (PET/CT- and MR-fused images) and contrasted enhanced pelvic MRI (ceMRI) had significantly higher T-staging accuracy (both 83.3%) than PET/CT (with contrast-enhanced CT, ceCT) 53.3% [84]. 

In cervical cancer, undiagnosed lymph node metastases present unique challenges during clinical staging. PET/MRI has shown promise as a non-surgical alternative for staging these metastases, offering advantages such as avoiding surgical risks, while reducing the time and costs associated with the procedure. Kim et al. evaluated 79 cervical cancer patients who underwent both MRI and PET/CT prior to lymphadenectomy. They found that tri-modality PET/MRI outperformed PET/CT in detecting regional lymph node metastases due to superior lymph node characterization via MRI [85]. Another study by Steiner et al. found that pre-treatment PET/MRI had a higher correlation between tumor size in imaging and pathology in patients with primary cervical cancer than did MRI alone (r_s_ = 0.87 vs. 0.58) [56]. In terms of N staging, PET/MRI and MRI were equally effective (areas under curve, AUC of 0.73). However, for M staging, PET/MRI performed better than MRI (AUC 0.80 vs. 0.67). This can be related to the higher specificity of PET/MRI (100% vs. 74%) [56].

For radiation treatment planning, PET accurately characterizes tumor volume and assesses lymph node status [86]. Combined with MRI, PET/MRI estimates tumor volume more accurately than PET/CT does due to the more precise identification of tumor margins via MRI [56]. PET/MRI improves treatment planning by providing a precise assessment of parametrial invasion compared with MRI alone (AUC 0.89 vs. 0.73) [56]. Acute therapy-induced edema and inflammation can lead to false-positive findings on post-treatment PET due to the increased uptake of FDG [87]. Advanced MRI techniques such as restriction spectrum imaging (RSI) may addresses this problem of false positives as edema may be distinguished from the residual tumor [88].

PET/MRI may be useful in distinguishing tumor recurrence from radiation-induced anatomical and tissue changes, such as fibrosis and scarring, during post-treatment assessment [89,90]. Schwarz et al. found that the 3-month post-treatment FDG-PET metabolic response is more prognostic of survival outcomes than the pretreatment lymph node status, with 3-year progression-free survival (PFS) rates of 78%, 33%, and 0% for complete- and partial-metabolic response, and progressive disease, respectively (*p* < 0.001) [91]. Moreover, Kidd et al. demonstrated pelvic lymph node SUV_max_, independent of the primary cervical tumor SUVmax, was a prognostic biomarker for treatment response, recurrence, and survival [76].

### 3.2. Endometrial Cancer

Studies have demonstrated that PET/MRI offers quantitative assessment data, aiding in the evaluation of disease extent and the selection of appropriate treatment plans for endometrial cancer [67,68,92]. Tsuyoshi et al. compared imaging biomarkers of pretreatment-integrated PET/MRI, using a reduced FOV (rFOV) DWI, between low- and high-risk endometrial cancers [92]. The SUV/ADC, characterizing tumor aggressiveness, demonstrated the greatest diagnostic accuracy compared with ADC and SUV alone (AUCs were 0.83, 0.72, and 0.66, *p* < 0.05, respectively). This finding can be valuable for selecting an appropriate treatment plan. Another study found a notable inverse correlation between SUV_max_ and ADC_min_ in 47 endometrial cancer patients who underwent integrated PET/MRI (r = −0.53; *p* = 0.001) [67]. Additionally, tumors of an advanced stage, and with deep myometrial invasion, cervical invasion, lymphovascular space invasion (LVSI), and lymph node metastasis exhibited significantly higher SUV_max_/ADC_min_.

Both MRI and PET/CT are useful in evaluating endometrial cancer (in addition to standard female pelvic ultrasound) [93]. The NCCN 2020 guidelines recommend MRI for initial locoregional assessment [94]. Whole-body PET/CT is used to assess lymph nodes and distant metastases in clinically suspected low-grade and all high-grade tumors [95,96,97]. For the post-therapy evaluation of clinically suspected recurrence, either a ceCT of the chest, abdomen, and pelvis or a whole-body FDG-PET/CT, along with an MRI of the pelvis or an MRI of the abdomen, are considered suitable options [31]. However, the combined modality of PET/MRI adds value when analyzing menstrual changes and during endometrial cancer staging.

**Figure 5:**
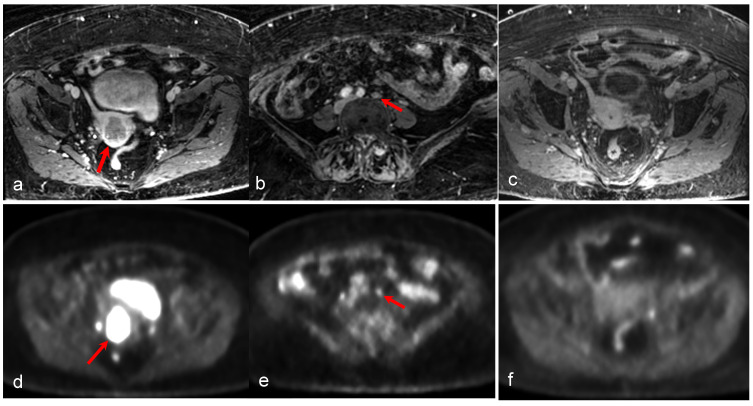
An 81-year-old woman with high-grade serous carcinoma of an endometrial origin. FDG PET/MRI was obtained for staging. Axial-focused PET image (**d**) demonstrating focal intense FDG uptake within the lower uterus and upper cervix, corresponding to a hypoenhancing mass seen on the axial T1-weighted post-contrast image (**a**). Additionally, an 8 mm short axis left common iliac chain node with mild FDG uptake was noted (**b**,**e**). There was no evidence of more distant metastatic disease. The patient was treated with pelvic radiation including boost to the left iliac chain lymph node, and subsequent chemotherapy. Follow-up axial T1-weighted image (**c**) and PET/MR (**f**) revealing complete metabolic response with absent FDG uptake within the mass, and complete resolution of abnormal enhancement with only a small amount of non-enhancing fluid in the endometrial canal. (Courtesy of Eric C. Ehman, MD, Department of Radiology, Mayo Clinic, Rochester, MN, USA).

**Figure 6:**
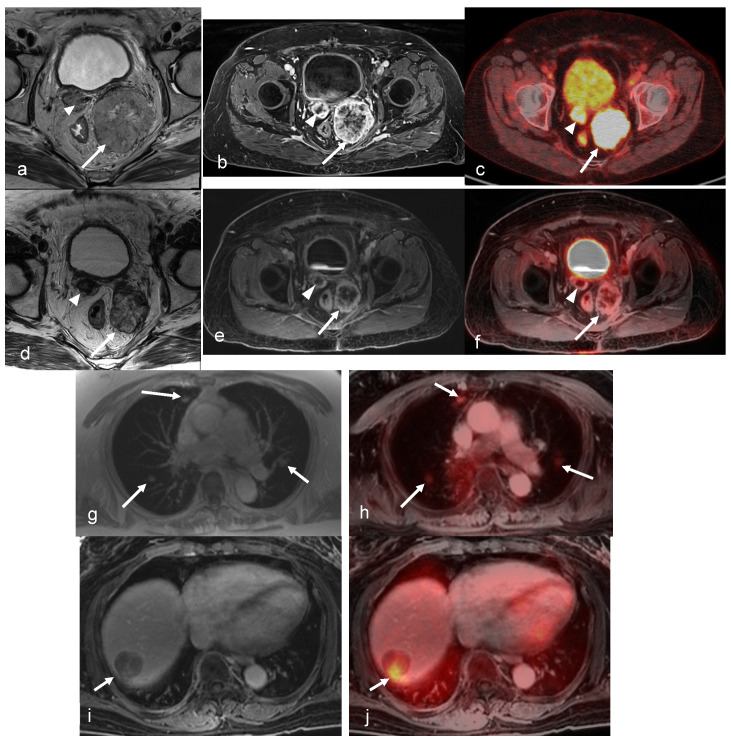
A 68-year-old female’s history of stage IVb endometrioid endometrial cancer who underwent systemic therapy, hysterectomy, and bilateral salpingo-oophorectomy with recurrent disease and pelvic implants in the vaginal cuff. Axial oblique small FOV T2-weighted (**a**) and axial post-contrast T1-weighted fat-saturated images (**b**) show two T2 intermediate signals, heterogeneously enhancing pelvic implants in the vaginal cuff (arrow and arrowhead). Fused PET/CT (**c**) images show intense FDG uptake in the pelvic implants (arrow and arrowhead). After external beam radiation therapy and brachytherapy, axial oblique small FOV T2-weighted (**d**) and axial post contrast T1-weighted fat-saturated images (**e**) show a decrease in size and the enhancement of the two pelvic implants in the vaginal cuff (arrow and arrowhead). Fused PET/MRI images (**f**) show a decrease in FDG uptake in the pelvic implants (arrow and arrowhead) with the small residual rim of a viable tumor, compatible with the partial local treatment response. (**g**) The axial in-phase image of the chest shows three new pulmonary nodules, most likely metastasis (arrowheads). A fused PET/MRI image (**h**) shows FDG uptake in the pulmonary nodules (arrowheads). (**i**) An axial post-contrast T1-weighted fat-saturated image shows a new heterogeneously enhancing metastatic liver mass with focal intense FDG uptake on fused PET/MRI images (arrowhead in (**j**)). (Courtesy of Vipul Sheth, MD, PhD and Negaur Iranpour, MD, Department of Radiology, Stanford University, Stanford, CA, USA).

PET/MRI plays a crucial role in endometrial cancer staging by facilitating precise evaluation of myometrial invasion and lymph node involvement, both of which are vital for the accurate staging of the disease (Figure 5). Tsuyoshi et al. demonstrated that non-contrast-integrated PET/MRI had comparable performance to that of ceMRI for T staging and to that of ceCT for N and M staging [98]. The sensitivities of PET/MRI and ceCT for detecting regional nodal metastasis were 100% and 14.3%, respectively. Kitajima et al. compared tri-modality PET/MRI with PET/CT (ceCT) and reported that PET/MRI exhibited significantly higher accuracy for T staging (80% vs. 60%) and comparable accuracy for N staging in endometrial cancer patients [99]. Ironi et al. found that integrated PET/MRI had 77% accuracy in detecting myometrial invasion (MI) with a positive predictive value (PPV) of 89%, and 91% accuracy with a high negative predictive value (NPV) 96% in detecting lymph nodes [100]. The study also revealed that volume-derived MRI variables, such as volume index (VI), total tumor volume (TTV), and tumor volume ratio (TVR), as well as PET parameters (e.g., MTV and TLG), were significant predictors of LVSI. Furthermore, these volume-derived MRI variables were found to be accurate predictors of the risk group (high-risk vs. low-risk). In another study conducted by Bian et al., integrated PET/MRI was observed to be more accurate than PET/CT in detecting myometrial invasion (81.8% vs. 45.9%) [58]. Additionally, PET/MRI showed higher sensitivity and specificity in detecting regional lymph node metastases compared with PET/CT (sensitivity: 50% vs. 33.3% and specificity: 100% vs. 91.2%, respectively). All of these technologies are improved compared with standard female pelvic ultrasound, where the sensitivity, specificity, and accuracy of finding myometrial invasion ≥50% were 65.6%, 80.3%, and 75.8% [101]. Therefore, PET/MRI can be considered an alternative diagnostic strategy to conventional imaging modalities for preoperative staging, particularly for patients who are unable to receive contrast agents. PET/MRI can also be helpful in identifying recurrent disease during post-treatment assessment (Figure 6).

The combined modality of PET/MRI circumvents challenges associated with FDG uptake due to menstrual changes. The premenopausal endometrium may have physiologically low FDG uptake, with two peaks of high FDG uptake during each menstrual cycle: one during the first three days of menstruation and another mid-cycle [25,26,27,102,103,104,105]. Two potential explanations for these peaks are peristaltic motions of the sub-endometrial myometrium and endometrial degeneration/narcotization [106,107,108]. Thus, elevated FDG uptake in the endometrium adjacent to a cervical cancer region does not always indicate endometrial tumor invasion [27]. Physiologic FDG uptake can be distinguished from abnormal uptake via PET/MRI through a comparison of PET and MR images (Figure 3). 

### 3.3. Ovarian Cancer

While ultrasound and CT are often the primary imaging techniques for detecting malignant ovarian tumors, pelvic MRI and PET/CT are utilized during staging. In the case of advanced epithelial ovarian cancer, complementary whole-body staging is recommended. PET/MRI is helpful in the evaluation of ovarian lesions, TNM staging, the identification of patients who are not candidates for optimal surgery, and the differentiation of malignant FDG-avid lesions from benign ones.

**Figure 7:**
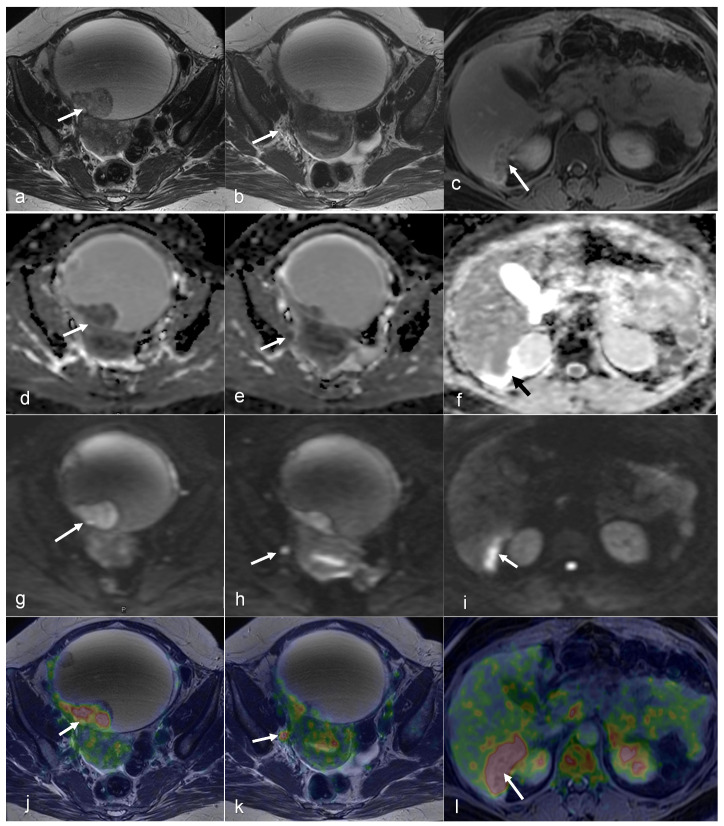
A 50-year-old female patient with peritoneal carcinomatosis secondary to ovarian clear cell carcinoma has a surgical peritoneal cancer index (PCI) score of 14 and a minimal amount of free fluid. On an axial T2-weighted image (**a**), a large cystic mass (hyperintense lesion) with a solid component appears slightly hypointense (arrow on (**a**)), shows restricted diffusion on the DWI and ADC map (arrows on (**g**,**d**)), and has high FDG uptake on fused PET/MRI (arrow in (**j**)). A 5 mm lymph node near the right internal iliac vessels is visibly hypointense on T2WI (arrow on (**b**)), shows restricted diffusion on DWI and ADC map (arrows in (**h**,**e**)), and has high FDG uptake (arrow on (**k**)). Notably, there is a peritoneal implant in the dorsal right liver lobe (arrows in (**c**,**f**,**i**,**l**)). (Courtesy of Elisabeth Hedlund, MD, Håkan Ahlström, MD, and Björg Jónsdóttir, MD, PhD, Uppsala University, Uppsala, Sweden).

PET/MRI has demonstrated higher sensitivity and specificity in the assessment of ovarian lesions (Figure 7), compared with PET/CT [109]. In a retrospective study, the findings indicated that PET/MRI had higher sensitivity (94%) and specificity (100%) compared with PET/CT (sensitivity: 74%, specificity: 80%) and MRI (sensitivity: 84%, specificity: 60%) [109]. Furthermore, the NPVs for PET/MRI, PET/CT, and MRI were 83%, 44%, and 50%, respectively. The PPVs were 100%, 93%, and 89%, respectively. Additionally, the diagnostic accuracy of PET/MRI using TNM staging is comparable with that of PET/CT, while showing no differences in detecting regional lymph node involvement and abdominal metastases [12]. Another challenge in ovarian cancer patients with carcinomatosis is estimating the total tumor burden since this is crucial for the decision of whether primary surgery should be suggested or not. PET/MRI has demonstrated superior accuracy to that of MRI in determining the peritoneal cancer index (PCI) in patients with a high tumor load and in defining patients not suitable for optimal surgery [101].

**Figure 8:**
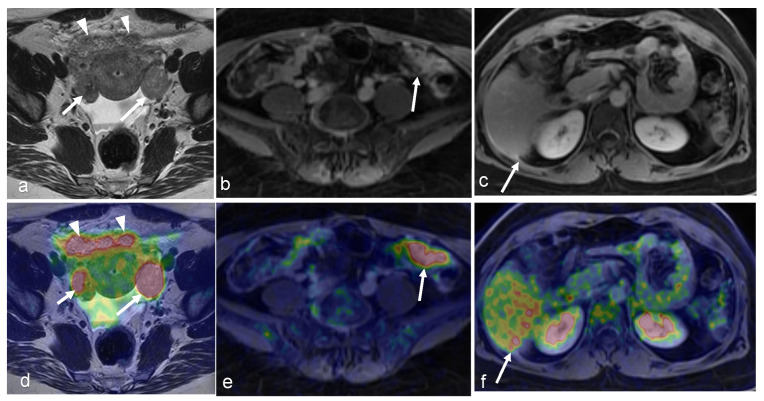
A 43-year-old woman with peritoneal carcinomatosis secondary to high-grade serous carcinoma has a surgical PCI score of 22. On an axial T2-weighted image, the bilateral ovarian tumors appear moderately hypointense (arrows in (**a**)) and show uptake of FDG on fused PET/MRI (arrows in (**d**)). Large omental caking ventral to the uterus and ovaries is also visible (arrowheads on (**a**,**d**)). Spread of the peritoneal implant to the left the paracolic gutter (in the lower abdomen (arrows in (**b**,**e**)) and the medial border of the right liver lobe can be observed (arrows in (**c**,**f**)) (Courtesy of Elisabeth Hedlund, MD, Håkan Ahlström, MD, and Björg Jónsdóttir, MD, PhD, Uppsala University, Uppsala, Sweden).

**Figure 9:**
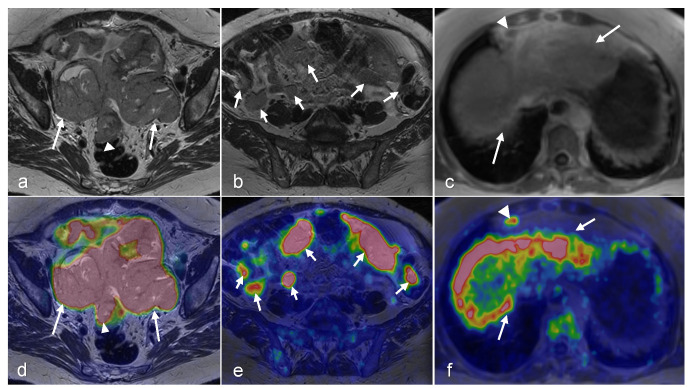
A 63-year-old woman with peritoneal carcinomatosis secondary to bilateral high-grade serous carcinoma with a PCI score of 39 with invasion of the rectal wall (arrowhead in (**a**,**d**)). On axial T2-weighted images, massive infiltration of the greater omentum is visible (arrows in (**a**,**b**)) with high FDG uptake (arrows in (**d**,**e**)) on axial-fused PET/MRI images. Both axial T1-weighted in-phase (**c**) and axial-fused PET/MRI (**f**) demonstrate multiple peritoneal implants spread across the surface of the liver, carcinomatosis implants on the diaphragm (arrows in (**c**,**f**)), and one supradiaphragmatic lymph node metastasis (arrowhead in (**c**,**f**)). (Courtesy of Elisabeth Hedlund, MD, Håkan Ahlström, MD, and Björg Jónsdóttir, MD, PhD, Uppsala University, Uppsala, Sweden).

PET/MRI may also be useful to overcome limitations of PET in ovarian lesions (Figure 8 and Figure 9), particularly with FDG uptake in non-malignant pathologies. Focal ovarian FDG uptake in women of reproductive age may be physiological rather than pathological [25,26,27]. Researchers have observed oval-shaped FDG uptake during the late follicular to early luteal phase, with an SUV greater than 3.0 [25,26]. During the luteinizing hormone (LH) peak, an increase in energy is required to grow a dominant follicle. In addition, there is a surge of macrophages and the production of numerous cytokines. Thus, corpus luteum formation following ovulation (Figure 2) is an inflammatory reaction that leads to a significant accumulation of FDG in macrophages [15,110]. However, in postmenopausal women, normal ovaries are non-FDG-avid, and any uptake in the ovaries or adnexa warrants further evaluation [27]. With the addition of MR, these non-malignant pathologies can be stratified while still receiving the benefit of PET in ovarian lesions. 

### 3.4. Vaginal and Vulvar Cancers

The ACR recommends MRI for locoregional assessment and PET/CT for the evaluation of nodal and distant metastatic involvement in the pretreatment assessment of recurrent vaginal cancer [111]. In addition, imaging is used for radiation planning to protect the surrounding healthy tissue from being irradiated. 

**Figure 10:**
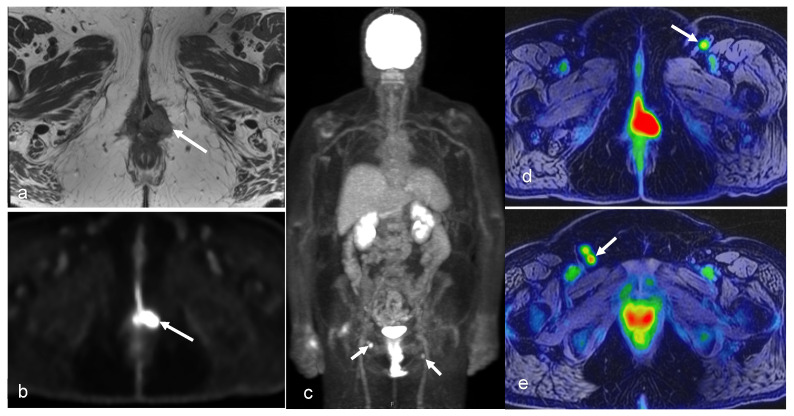
A 64-year-old woman with history of vulvar cancer diagnosed 4 years prior and initially treated with left vulvectomy. The patient was re-evaluated due to new symptoms of pain and itching, and a biopsy was performed, revealing a recurrent high-grade squamous neoplasm. PET/MRI was ordered for restaging. Axial PET (**b**) and T2-weighted (**a**) images from dedicated pelvis MRI with dedicated pelvic PET show an FDG-avid, nodular, intermediate T2 signal in the left perineum compatible with local recurrence. The whole-body survey MIP image (**c**) and axial-fused PET/MRI images (**d**,**e**) demonstrate bilateral FDG-avid inguinal lymph nodes without more distant metastatic disease. The patient went on to undergoing wide local excision with adjuvant radiation and chemotherapy. (Courtesy of Eric C. Ehman, MD, Department of Radiology, Mayo Clinic, Rochester, MN, USA).

PET/MRI can provide significant value in evaluating vaginal and vulvar tumors. For instance, PET/MRI can aid in distinguishing between recurrent disease and post-treatment or postsurgical changes [90] (Figure 10). Lymph node metastasis is the most important prognostic factor in vulvar cancer, despite the limited progress in the detection of lymph node involvement at an earlier stage over the past four decades [112]. According to Cohn et al., PET has high specificity but relatively low sensitivity and NPV in the detection of groin lymph node metastases arising from vulvar cancer [43]. In contrast, a retrospective study of 160 vulvar cancer patients assessed preoperative PET/CT for predicting groin and pelvic lymph node involvement [113]. PET/CT exhibited strong sensitivity and NPV, with a groin-level sensitivity of 78.9%, specificity of 78.2%, accuracy of 78.4%, PPV of 61.2%, and NPV of 89.4% [113].

## 4. PET/MR Considerations

### 4.1. Challenges

While PET/MRI offers significant advantages, it also poses unique challenges that require careful consideration. The physiologic activity of FDG by the bladder and intestines can potentially obscure the detection of pathological findings or lead to a false-positive diagnosis on PET/MRI of gynecologic malignancies. This can be reduced by correlating PET images with MR images. Another related challenge is high FDG uptake in non-malignant pathologies (e.g., infection and inflammation), potentially leading to false-positive PET findings [114]. Fortunately, dynamic contrast-enhanced (DCE) MRI and novel techniques such as DWI may aid in differentiation to overcome these challenges. 

Additionally, both sequential and integrated systems require significant modifications to PET and MR hardware and software to maintain similar performance to that of the standalone techniques (with most compromises on the MRI side). An example is the development of magnetic field-insensitive avalanche photodiodes and silicon photomultipliers (SiPMs) for the replacement of the conventional photomultiplier tubes of the PET detectors in integrated systems [115]. In general, the design and materials of PET and MR components are carefully considered to prevent negative impacts on PET from MRI and vice versa, including appropriate shielding designs to avoid eddy currents and using materials of low magnetic susceptibility to reduce susceptibility artifacts. 

The ability of using MRI for the attenuation correction of PET in sequential and integrated systems has been a topic of discussion. For adequate interpretation and quantification, PET data need correction for photon attenuation. This is because the photon count depends not only on the number of photons emitted but also on the linear attenuation coefficient (μ) and tissue thickness. Integrated and sequential PET/MR systems lack the ability for CT-based attenuation correction, so their attenuation maps (μ-maps) are estimated from MRI. T1-weighted or water–fat (i.e., Dixon) MR sequences are utilized for the generation of μ-maps in whole-body applications, including pelvic imaging. The images are typically segmented into up to four classes: air, soft tissue, fat, and lung tissue [116,117,118]. However, MR voxel intensities reflect proton density and MR relaxation properties, which are not directly convertible into μ-values. Instead, MR-derived μ-maps are created by assigning predefined μ-values (fixed or continuous) to the voxels of each class, rather than patient-specific values [117,118]. 

A challenge with the described MR-based attenuation correction (MR-AC) is its inability to visualize bone, metal implants, and MR hardware within the PET FOV, despite their significant photon attenuation. Bone is typically substituted with soft tissue, leading to an underestimation of bone attenuation and the SUV in regions within or near the skeleton [119,120,121]. In gynecologic imaging, the SUV quantification of lesions in the vicinity of the pelvic bone could be affected. To reduce such errors, there are two MR-AC methods that include bone for whole-body applications. The first method utilizes a deep neural network to segment bone, along with air, lung, fat, and lean tissue in the Dixon MR images [117]. The second method adds bone from a model-based bone segmentation algorithm to the Dixon-derived μ-map [121]. Additionally, there are specific alternatives available for the bone attenuation of the skull, such as ultrashort or zero-echo-time sequences [116,122]. In PET/MRI, metal implants give rise to artifacts on MRI, which degrade both the MR and PET images’ quality. The implants cause MR signal loss and artifacts due to their low proton density and strong magnetic susceptibility compared with adjacent tissue [117,123].

Due to MR hardware limitations (decreasing B_0_ homogeneity and gradient linearity with distance from the isocenter), the maximum achievable MR FOV is smaller than the PET FOV (~50 cm vs. ~60–70 cm), which might cause truncation artifacts in the MR-AC. Patients with arms positioned alongside the body and obese patients can give rise to such artifacts, affecting PET reconstruction in various body regions, including the pelvis [124]. This issue has been reduced to clinically acceptable levels via two main approaches: (1) B_0_ homogenization using gradient enhancement (HUGE), in which an optimal readout gradient field for the minimization of B_0_ inhomogeneities and gradient nonlinearities outside the MR FOV is determined [125]; (2) estimation of the contour of tissue outside the MR FOV from non-attenuation corrected PET images [117]. B_0_ homogeneity is also affected by metal implants, which cause MR signal loss and artifacts [123]. The affected image region often exceeds the implant size, leading to incorrect attenuation correction in its vicinity. Currently, no reliable and robust MR-based attenuation correction method, accounting for metal implants, is commercially available. The manufacturers have solved the issue of the attenuation of hardware (e.g., receiver coils) within the PET FOV by using predefined μ-maps from CT-based templates for fixed coils, and made efforts to displace highly attenuating material to outside the PET FOV [126,127]. It is recommended to always use the most current software version and attenuation correction protocols on the designated PET/MRI system due to the complexity and increasing variety of attenuation correction methods [128].

As reported from the International Workshop on PET/MRI in 2017, MR-AC is generally considered acceptable for most routine clinical situations, with uncertainties comparable to those of PET/CT [122]. However, the PET/MRI oncology community still requested efforts to reduce residual bias from MR-AC and truncation artifacts [122]. According to Eiber et al., both PET/CT and PET/MRI demonstrated a strong correlation in SUV estimation across various tumors (r = 0.9975, *p* < 0.0001) [129]. Variations can occur in measured tracer uptake between PET/MRI and PET/CT due to the utilization of distinct PET quantification methods in each modality. Thus, caution is advised when comparing results or conducting repeated examinations, such as those undertaken pre- and post-therapy. Consistently using the same modality is preferable for more comparable results.

A further challenge with PET/MRI is the high cost. The purchase price of the PET/MR scanner is more than that of standalone PET or MRI systems, and the former is not as widely available in healthcare facilities [130]. However, the ability of integrated and sequential PET/MR systems to produce multiparametric PET and MR images simultaneously, as well as to function as standalone MRI or PET methods when the other imaging outcomes are not required, can potentially aid in the establishment of this hybrid modality as a cost-effective imaging alternative in oncology [130]. Examples of such multiparametric images in gynecologic malignancies of the female pelvis are provided in Figure 1, Figure 2, Figure 3, Figure 4, Figure 5, Figure 6, Figure 7, Figure 8, Figure 9 and Figure 10.

### 4.2. Future Directions

A future prospect specific to integrated PET/MR systems, compared with tri-modality and sequential systems, is that the static magnetic field (B_0_) shortens the range of the positron, leading to an increased PET spatial resolution in the plane perpendicular to the direction of B_0_, compared with that of PET/CT [131]. The effect increases with increased magnetic field strength and positron energy. For the most common radiotracer FDG, the effect is minimal because of the relatively low energy of the emitted positron, but would be substantial for medium- and high-energy positrons (e.g., ^68^Ga and ^120^I, respectively) [132]. The development of novel tracers for gynecologic malignancies of the female pelvis, based on molecules with higher disease specificity compared to FDG and radionuclides with medium/high-energy positrons, has potential to increase the possibility to differentiate between malignant and surrounding tissue.

Due to considerable scan times, PET images can be influenced by motion (e.g., cardiorespiratory motion; movement of other internal organs) [133]. With gating techniques, artifacts from periodic motion are usually reduced by limiting the PET acquisition or reconstruction to a predefined phase, which results in a loss of valuable data and a decreased signal-to-noise ratio. This issue can be addressed via motion tracking followed by retrospective correction through image registration, or via prospective motion correction incorporated already at image reconstruction [133]. For such methods, simultaneously acquired MR data from integrated PET/MR systems provide anatomical details that can be used alone or in combination with PET for improved motion correction [134,135,136,137], as compared with sequential PET/CT [138]. MR-based retrospective correction for respiratory and/or cardiac motion has been implemented by PET/MR manufacturers [139,140,141]. Prospective motion correction has been used within research for oncological applications [134,142]. While motion correction has shown notable impacts in cancer imaging, particularly for lung and liver lesions, its implementation in clinical practice is limited and remains a prospect for integrated PET/MRI [133]. Methods for the correction of non-periodic motion, such as those associated with bladder filling, are also topics for future development [133]. In the PET/MRI of female pelvic malignancies, a whole-body PET is commonly acquired simultaneously with a whole-body MRI. The latter is used for both diagnostic purposes and as an anatomical reference to the PET images. A dedicated MRI of the pelvic region, consisting of multiple pulse sequences, is usually performed separately within the same examination. To improve the anatomical alignment between the whole-body PET and the dedicated pelvic MRI, any motion-related disparities (e.g., those arising from bladder filling) can be effectively addressed and corrected by using the MR data.

Artificial intelligence (AI) has emerged as a powerful tool in medical imaging. PET images often suffer from noise and limited spatial resolution. AI models, including convolutional neural networks, U-Nets, and generative adversarial networks, have demonstrated improvements in denoising and image enhancement [143,144,145]. These advancements can potentially reduce radiotracer doses and scan times, as well as improve workflow efficiency [144,145]. Deep learning methods can be utilized to transform MR images into pseudo-CT images, which are necessary for attenuation correction in PET/MRI [145]. However, challenges remain, including poor model generalizability, which may result in variable performance across different scanners or protocols [146]. Multidisciplinary collaborations between clinicians and AI experts are essential for the practical application of AI in routine clinical practice.

## 5. Conclusions

PET/MRI evaluation is becoming increasingly popular as it provides complementary physiological and molecular information from PET with anatomical and physiological information from MRI. In the case of female pelvic malignancies, FDG-PET/MRI has shown to be more accurate than FDG-PET/CT, in the assessment of staging (local tumor extent, lymphadenopathy, and extra pelvic metastases at diagnosis) and therapy evaluation. This multi-modality approach can help minimize false positives and/or false negatives, and consequently improve diagnosis and reduce the use of unnecessary treatments. Integrated PET/MR systems allow for simultaneous PET and MRI with benefits such as improved anatomical alignment between the modalities, reduced total scan time, and reduced doses of ionizing radiation. Future technological improvements, with respect to the development of novel tracers for female gynecological malignancies, MR-based attenuation correction, and motion correction, could further enhance the use of these systems. To justify the higher expenses associated with PET/MRI, it is necessary to conduct research demonstrating the impact of this combined modality on patient management and the improvement in outcomes of pelvic malignancies. Overall, PET/MR imaging has the potential to become a valuable tool in the clinical management of pelvic malignancies and may offer several advantages over other imaging modalities.

## Figures and Tables

**Table 1:** Benefits and pitfalls of imaging modalities in gynecologic malignancies.

Malignancy		CT	MRI	PET/CT	PET/MRI
Cervical	Benefit(s)	Evaluation of regional lymph nodes, distal metastases, hydronephrosis [19]	High diagnostic accuracy for local staging and assessing primary tumor and pelvic lymph node metastasis, defining advanced disease Helpful in treatment planning, monitoring treatment response, and post-treatment surveillance to detect local recurrence [20,21,22,23]	Detection of primary tumor, assessment of tumor volume, lymph node, and distant metastases [23] Assessment of treatment response and tumor recurrence [19]	Excellent performance in the evaluation of stage, regional and distant nodal involvement, and metastatic disease Simultaneous soft tissue and metabolic assessment [19]
	Pitfall(s)	Limited in assessment of cervical tumor invasion, parametrial invasion, and pelvic sidewall involvement Limited in evaluation of micro-metastatic disease in lymph nodes < 1 cm Cannot reliability detect reactive nodes versus metastatic nodes > 1 cm [19]	Limited in evaluation of micro-metastatic disease in lymph nodes [19] Cannot reliability detect reactive nodes versus metastatic nodes. Limited in differentiating between tumor recurrence and post-treatment inflammatory changes [24]	The physiological FDG uptake in the premenopausal endometrium adjacent to cervical cancer can be mistaken for endometrial tumor invasion [25,26,27] False positive FDG uptake during benign conditions (e.g., infection) and post-therapy changes can mimic malignancy [19]	Less sensitive for detection of pulmonary nodules compared with PET/CT [13]
Endometrial	Benefit(s)	Routinely used in evaluation of patients to identify metastatic disease within the lungs and lymph nodes [24]	Accurate modality for local staging, tumor delineation, assessment of myometrial invasion and pelvic lymphadenopathy, defining advanced disease [27,28] Helpful in planning treatment, monitoring treatment response, and post-treatment surveillance [29]	Diagnostic tool for staging and surveillance of cancer Detecting positive pelvic and/or para-aortic lymphadenopathy and distant metastasis [29]	Staging of nodal and distant metastases during local staging Simultaneous soft tissue and metabolic assessment. [25]
	Pitfall(s)	Limited in evaluation for local staging Difficult to assess the vaginal vault [24] Overestimating the central tumor volume due to the presence of tissue reaction and edema near the tumor–tissue interface [30]	Overestimating the tumor volume due to the presence of post treatment edema of the tumor [30]	Routine use is not recommended in preoperative staging in early stage disease as 45% of endometrial cancers are not FDG-avid [31]	Less sensitive for detection of pulmonary nodules compared with PET/CT
Ovarian	Benefit(s)	Evaluates for metastatic disease and possible lymph node involvement. Useful for determining response to chemotherapy, can predict diaphragm and omental involvement [32]	Outperforms CT and PET/CT for detecting ovarian cancer [33] Helps differentiate between benign, malignant, and borderline masses by DCE-MRI and DWI [34] Useful for treatment planning in advanced ovarian cancer [32]	Evaluating possible metastatic extraperitoneal spread of the disease and metastatic lymph nodes [32] Detects recurrent disease [32] predicts treatment response after NAC [35,36,37,38,39,40]	Hybrid molecular and anatomic imaging provides high soft tissue contrast with lower radiation dose Detects lymph node metastases with high accuracy [32]
	Pitfall(s)	Limited soft tissue evaluation and differentiation. Limited in evaluating local extent of disease	Limited sensitivity in detecting small peritoneal implants [41]	Lack of reliable differentiation between borderline and benign tumors according to ESGO/ISUOG/IOTA/ESGE Consensus Statement on pre-operative diagnosis of ovarian tumors. No clear cut-off value for maximum standardized uptake value for differentiation between benign and malignant ovarian tumors [32] Not recommended for primary detection of ovarian cancer [32] The physiologic FDG uptake in pre-menopausal ovaries can be mistaken with malignancy [25,26,27]	Less sensitive for detection of pulmonary nodules compared with PET/CT
Vaginal/Vulvar	Benefit(s)	Helpful in determining disease extent and nodal/metastatic involvement [42] Useful for identifying distant metastases, including pulmonary and bony metastases in vulvar cancer [42]	The modality of choice for locoregional assessment, detection of primary and metastatic cancer, and treatment response The most sensitive modality for detecting pelvic lymph node involvement [42]	Useful for radiation therapy planning [43], assessing response to neoadjuvant chemotherapy and guide patient management Evaluation of nodal and distant metastatic involvement in staging of recurrent vaginal cancer [42]	Helpful in for detecting vulvar cancer recurrences and distant metastases [42]
	Pitfall(s)	Difficulty in assessing lymph node involvement, especially in small or micro-metastatic nodes Inability to determine local tumor staging due to low soft tissue contrast [42]		Limited value in detecting lymph node metastases ≤ 5 mm and necrotic lymph nodes False-positive (e.g., inflammatory lymph node) [23]	

**Table 2:** PET/MR Systems.

Design	Description	Advantages	Disadvantages
Tri-modality [44,45] 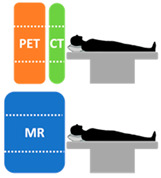	Separate PET/CT and MR systems Shared transport bed, compatible with both scanners, improves anatomical correspondence between PET and MRI	Relatively low cost Access to image data from three modalities (including CT-based attenuation correction of PET data) Flexibility to use the systems independently	Risk of misalignment due to patient motion or bowel motility Longer examination time compared to sequential and integrated systems
Sequential [46] 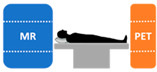	PET and MR bores positioned in a serial fashion MR images acquired immediately after PET, within the same examination	Reduced dose of ionizing radiation by not conducting a CT Reduced total examination time Lower likelihood of image misalignment compared to tri-modality systems due to the shorter time between scans	Special shielding and additional space requirements due to systems proximity Lack of conventional CT-based attenuation correction Potential impact on the quality of reconstructed PET images due to MR-based attenuation correction
Integrated [47] 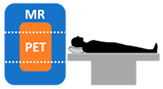	Simultaneously acquiring PET and MR images by incorporating PET into the MR bore	True simultaneous PET and MRI acquisitions Improved image alignment between modalities Reduced dose of ionizing radiation Reduced total examination time	High purchase price compared to sequential systems Lack of CT-based attenuation correction The mutual negative impact of PET and MR hardware

## Data Availability

All data relevant to this study are contained within the manuscript. No additional data beyond those presented in the manuscript can be provided.
